# Ultrasound biomicroscopy analysis of ciliary muscle dynamics and its relation to intra-ocular pressure after phacoemulsification in dogs

**DOI:** 10.3389/fvets.2024.1366997

**Published:** 2024-05-09

**Authors:** Donghee Kim, Sang-Eun Park, Jiyi Hwang, Nanyoung Kang, Ji Seung Jung, Kyung-Mee Park

**Affiliations:** Laboratory of Veterinary Surgery and Ophthalmology, College of Veterinary Medicine, Chungbuk National University, Cheongju, Republic of Korea

**Keywords:** ciliary cleft, ciliary body, ciliary muscle, phacoemulsification, glaucoma, ultrasound biomicroscopy, aqueous humor, cataract

## Abstract

**Introduction:**

This study investigates the relationship between ciliary muscle dynamics, thickness, and the regulation of intraocular pressure (IOP), focusing on the progression of cataracts and changes post-phacoemulsification. It explores how these factors impact canine ocular health, particularly in the context of cataract development and subsequent surgical intervention.

**Materials and methods:**

Data was collected using Ultrasound Biomicroscopy (UBM) from dogs at the Veterinary Medical Teaching Hospital of Chungbuk National University, Korea. The study involved 57 eyes from 35 dogs, categorized into five groups: 13 normal eyes, 14 with incipient cataracts, 12 with immature cataracts, 6 with mature cataracts, and 12 post-phacoemulsification. UBM measurements assessed various ciliary muscle parameters including ciliary body axial length (CBAXL), ciliary process-sclera angle (CPSA), longitudinal fibers of ciliary muscle thickness (Lf-CMT), and longitudinal and radial fibers of ciliary muscle thickness (LRf-CMT).

**Results:**

Findings indicated a decrease in CBAXL and an increase in Lf-CMT as cataracts progressed in severity. Post-phacoemulsification, there was a notable increase in CBAXL and a decrease in CPSA, Lf-CMT, and LRf-CMT, compared to both cataractous and normal eyes. Regression analysis revealed a significant positive association between CBAXL and IOP, alongside a negative association between Lf-CMT and IOP. These findings suggest that variations in ciliary muscle dynamics and thickness, as influenced by cataract progression and phacoemulsification, have distinct impacts on intraocular pressure.

**Discussion:**

The study proposes that phacoemulsification leads to ciliary muscle contraction, causing an inward and anterior movement of the ciliary muscle. This movement results in the narrowing of the ciliary cleft and constriction of the unconventional outflow pathway, potentially causing an increased risk of glaucoma post-surgery. Our research contributes to understanding the anatomical and physiological changes in the canine eye following cataract surgery and underscores the importance of monitoring IOP and ciliary muscle dynamics in these patients.

## 1 Introduction

The ciliary muscle plays a crucial role in regulating the flow of aqueous humor (AH). Extensive research has been conducted in human medicine to examine the impact of ciliary muscle contraction and relaxation on AH flow (1). It is well-established that the tone of the ciliary muscle influences the unconventional outflow pathways (2, 3). The ciliary muscle serves as a common entry point for the uveoscleral and uveovortex routes, which are considered significant in unconventional outflow pathway (2). The ciliary muscle acts as a rate-limiting step in these pathways (4).

Additionally, the anterior tendons of the ciliary muscle are connected to the trabecular meshwork, which is essential for the conventional outflow pathway. Upon contraction of the ciliary muscle, the anterior tendons move anteriorly and inwardly, resulting in the widening of the trabecular meshwork and dilation of Schlemm’s canal in humans. These actions ultimately reduce the resistance to AH outflow (4, 5).

Glaucoma is a common adverse outcome that occurs after cataract surgery in dogs. In a recent study, 88 out of 505 study eyes developed glaucoma during their follow-up period after phacoemulsification in dogs (6). In our previous study, it was found that the collapse of the ciliary cleft (CC) is a contributing factor to the development of glaucoma after phacoemulsification (7). The CC is a dynamic structure that contracts or expands depending on IOP, and it serves as a common entrance for both the conventional and unconventional outflow pathways (8). The collapse of the CC after phacoemulsification can hinder the smooth outflow of AH (9). While the exact mechanisms behind this phenomenon remain uncertain, research in human medicine provides plausible hypotheses regarding its causes. In studies on presbyopia in humans, it is acknowledged that after phacoemulsification, the restoration of the ciliary muscle’s contractile force leads to the inward movement of the ciliary body (10). Extending this understanding to canines, it is posited that the rejuvenation of contractility in the ciliary muscle following phacoemulsification subsequently exerts pressure on the ciliary cleft. As a result, this inward contraction of the ciliary muscle is thought to play a significant role in the collapse of the ciliary cleft.

Research in human medicine has shed light on the biomechanical effects of cataract formation and subsequent phacoemulsification surgery on the eye’s structure. The process of cataract formation leads to the lens hardening, which in turn increases the tension within the zonules. This heightened tension is responsible for the relaxation of the ciliary muscle among patients with cataracts. In contrast, the surgical removal of cataracts through phacoemulsification has been shown to reduce zonular tension. This reduction facilitates the restoration of contractility in the ciliary muscle (10, 11). The contraction of the ciliary muscle after phacoemulsification results in anterior and inward movement of the muscle. This movement also leads to thinning of the longitudinal bundle fibers of the ciliary muscle (12, 13). The impact of these dynamic changes in ciliary muscle morphology on the AH pathway is well-documented in human medicine (14, 15). However, because of anatomical differences in the iridocorneal angle between dogs and humans, it is difficult to directly apply these findings to dogs.

The objective of this study was to assess the structural changes that impact the AH flow by examining the ciliary muscle before and after phacoemulsification in dogs. The ultimate aim is to identify the underlying possible cause of glaucoma that occurs following phacoemulsification. For this, the morphology and thickness of the ciliary muscle were examined before and after the phacoemulsification. We also investigate the influence of morphology on the collapse of the CC and the impact of ciliary muscle thickness on the unconventional outflow pathway, considering the IOP.

## 2 Materials and methods

### 2.1 Clinical information

The clinical data of dogs included in this study was collected from the Veterinary Medical Teaching Hospital of Chungbuk National University in Korea, spanning from August 29th, 2018, to September 20th, 2022. A total of 57 eyes (35 dogs) were examined. The Institutional Animal Care and Use Committee (CBNUA-1700-22-02) granted approval for this research. The ophthalmological examinations were conducted by a veterinary faculty member named KMP and other veterinarians specialized in ophthalmology. Various tests were performed during the examination, including slit-lamp biomicroscopy (MW50D, SHIGIYA, Hiroshima, Japan), the Schirmer Tear Test (Schirmer Tear Flow Strips, GuldenOphthalmics, PA), menace response, pupillary light reflex, dazzle reflex, rebound tonometry (TonoVet plus^®^, icare, Vantaa, Finland), gonioscopy (Ocular Koeppe Diagnostic Lenses, Ocular Instruments.inc, Bellevue, WA), indirect ophthalmoscopy (Pan Retinal^®^ 2.2, VOLK, OH), Electroretinogram (RETevet^®^, LKC technologies, MD) and UBM (VuPAD^®^, Sonomed Escalon, Lake Success, NY). The cataract stage was classified based on previous studies as incipient, immature, mature, or hypermature.

#### 2.1.1 Patient groups

The dogs in this study were categorized into three groups: (a) normal eyes (*n* = 13), (b) cataracts (*n* = 32), and (c) post-phacoemulsification (post-phaco; *n* = 12). In this study, when a patient’s eyes belonged to different groups, one eye was randomly selected for inclusion to avoid statistical errors (16). To facilitate further analysis, the normal eye and cataract groups were further divided into two subgroups: (a) Surgical Treatment Candidate (STC) group (*n* = 18) and (b) Non-Surgical Management (NSM) group (*n* = 27) (17–22). The STC group included dogs with immature and mature cataracts, while the NSM group comprised normal eyes and dogs with incipient cataracts.

#### 2.1.2 Inclusion criteria

Patients with hereditary or senile cataracts were eligible for inclusion in this study. Only patients who had a follow-up period of at least 2 months (with a mean duration of 662 ± 465.14 days and a range of 61 to 1,365 days) after undergoing phacoemulsification were included in the analysis of post-phaco outcomes.

#### 2.1.3 Exclusion criteria

In this study, subjects with systemic diseases were excluded. Additionally, patients presenting abnormalities such as uveitis, glaucoma, or hemorrhage prior to surgery were not included in this study, with the exception of those affected by cataracts. Furthermore, cases showing abnormal results in the electroretinogram (ERG) were also excluded from this research. Patients with secondary cataracts caused by diabetes or trauma were not considered for inclusion. Eyes demonstrating a closed iridocorneal angle (ICA), as detected during gonioscopy, and patients who had received a Capsular Tension Ring (an-CTR-12, An-vision, Hennigsdorf, Germany) were additionally excluded from the analysis.

### 2.2 UBM examination

UBM was performed during the ophthalmic examination in both normal and cataractous eyes in this study. In post-phaco eyes, UBM was conducted at least 2 months after cataract surgery. The dogs were positioned in a sitting posture, and their pupils were dilated by applying 0.5% tropicamide topically (Mydriacyl^®^; Alcon, Geneva, Switzerland). Additionally, topical anesthesia was administered using one drop of 0.5% proparacaine hydrochloride (Alcaine^®^; Alcon). The palpebral fissure was manually opened, and the eye was examined by placing a transducer perpendicularly to the corneoscleral limbus in the dorsal quadrant.

The study included measurement of the following parameters: axial length of the ciliary body (CBAXL), ciliary process-sclera angle (CPSA), longitudinal fiber of ciliary muscle thickness (Lf-CMT), and longitudinal and radial fiber of ciliary muscle-choroid thickness (LRf-CMT) according to a previous report (8, 10, 11, 13, 23–25).

**Figure 1:**
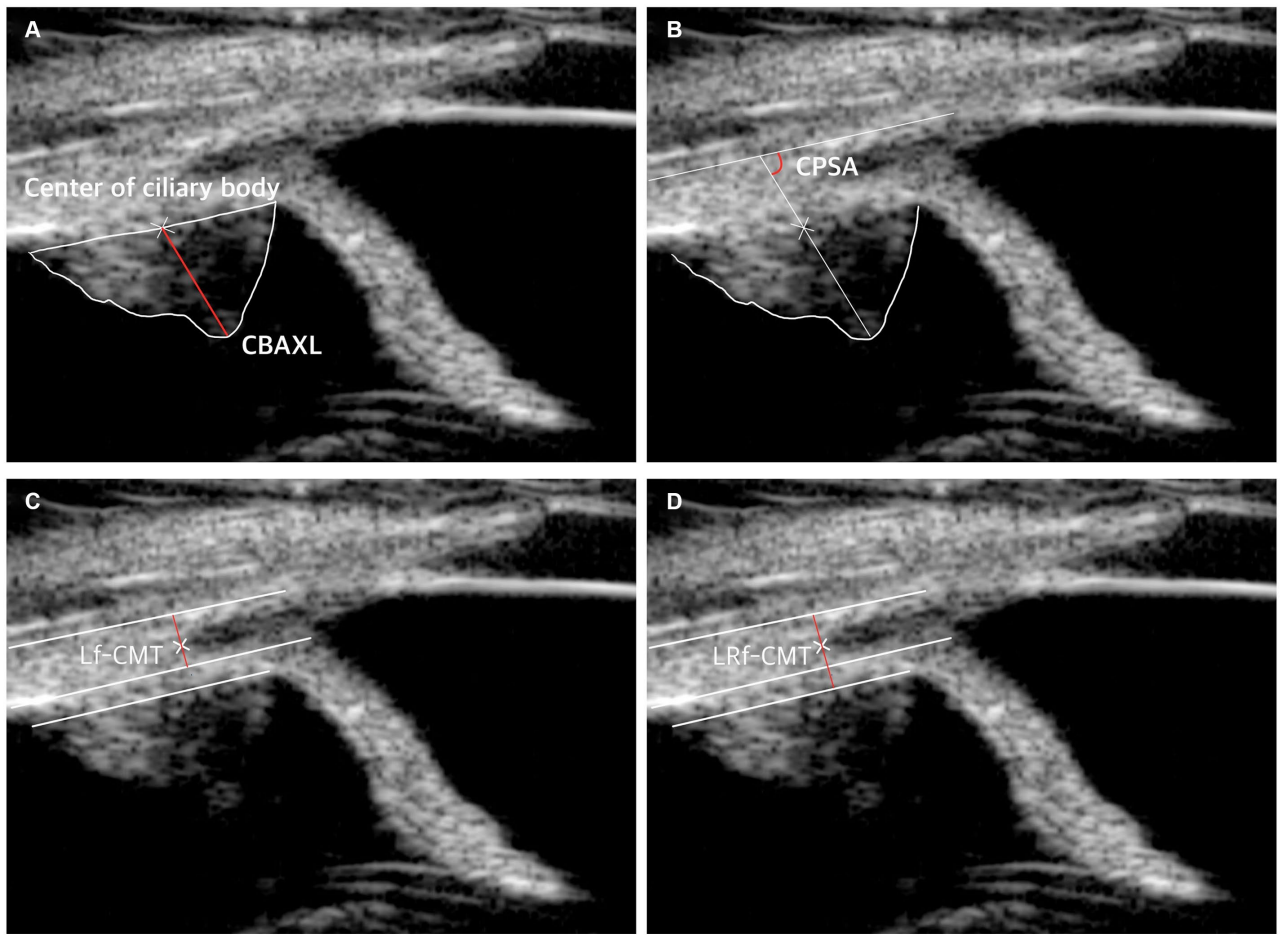
UBM measurement method. **(A)** Measurement of axial length of the ciliary body (CBAXL). A line was drawn through the apex of the dome-shaped ciliary body and the center of the ciliary body to calculate CBAXL. **(B)** Measurement of ciliary process-sclera angle (CPSA). CPSA was defined as the angle between the long axis of the ciliary body and the sclera. **(C)** Measurement of longitudinal fiber of ciliary muscle thickness (Lf-CMT). A line was drawn through specific points to determine Lf-CMT, including the inner layer of the sclera, the angle recess, and a line parallel to the inner layer of the sclera starting from the point where the inner layer of the iris root meets the pectinate ligament. **(D)** Measurement of longitudinal and radial fiber of ciliary muscle-choroid thickness (LRf-CMT). LRf-CMT involved drawing a line through the inner layer of the sclera, the angle recess, and a line parallel to the inner layer of the sclera starting from the point where the outer layer of the iris root meets the dome-shaped ciliary body.

To determine CBAXL, a line was drawn through the apex of the dome-shaped ciliary body and the center of the ciliary body. This measurement was performed manually using the built-in software of UBM. CBAXL was calculated as the distance between the apex of the ciliary body and the uveoscleral interface along the long axis of the ciliary body (Figure 1A). CPSA was defined as the angle between the long axis of the ciliary body and the sclera (Figure 1B). For the measurement of Lf-CMT, a line was drawn through the inner layer of the sclera, the angle recess, and a line parallel to the inner layer of the sclera starting from the point where the inner layer of the iris root meets the pectinate ligament (Figure 1C). The measurement of LRf-CMT involved drawing a line through the inner layer of the sclera, the angle recess, and a line parallel to the inner layer of the sclera starting from the point where the outer layer of the iris root meets the dome-shaped ciliary body (Figure 1D) (8, 13, 23–25).

### 2.3 Cataract surgery: pre and post-surgical management

Prior to the surgery, the dogs received pre-operative treatment with specific medications. They were administered topical ophthalmic antibiotics (moxifloxacin, Vigamox^®^, NOVARTIS, East Hanover, NJ or ofloxacin, OcuFlox^®^, Samil, Seoul, Korea), non-steroidal anti-inflammatory drugs (flurbiprofen, flurbiprofen^®^, Bausch & Lomb, Bridgewater, NJ, or bromfenac, Bronuck^®^, TAEJOON PHARM, Seoul, Korea), and steroids (neomycin-polymyxin B sulfate-dexamethasone Maxitrol^®^, NOVARTIS, East Hanover, NJ or prednisolone acetate 1%, Pred-Forte^®^, Allergan, North Chicago, IL) based on a specific schedule: q8h, q12-24 h, and q8h, respectively, for a duration of 5 days. Moreover, before the surgery, the dogs received a different pre-operative treatment involving amoxicillin-clavulanate (AMOCLA^®^, KUHNIL PHARM, Cheonan, Korea), famotidine (Famotidine, Nelson, Eumsung, Korea), and carprofen (ASHICARP^®^, ALS, Mumbai, India) for a period of 5 days. Additionally, just 3 to 4 h before the surgical procedure, the dogs received a specialized ophthalmic solution in three to four cycles, with 5–10 min intervals between cycles. This solution included moxifloxacin or ofloxacin, flurbiprofen or bromfenac, neomycin-polymyxin B sulfate-dexamethasone or prednisolone, tropicamide, and atropine (Atropine^®^, Alcon) for each eye.

The phacoemulsification procedure was conducted by a veterinary faculty member (KMP) and two veterinarians specializing in ophthalmology (JHA and JWP) utilizing three different machines: Stellaris PC (Bausch & Lomb Inc.), Pulsar Minimal Stress (OPTIKON, Roma, Italy), or Sovereign Compact Phaco Machine (AMO, Andrew Place Santa Ana, CA). Foldable acrylic IOLs (Loki^®^, CRISTALENS, Lannion, France) with a power of +41.0 diopters and a diameter ranging from 12.00 to 14.00 mm were carefully implanted through a 3.0 mm stepped clear corneal incision. Prior to the surgical procedure, the irrigation fluid was prepared by adding additives of epinephrine (1 mg/mL; DAIHAN, Seoul, Korea) and heparin (5,000 units/mL; JW, Seoul, Korea) to a balanced salt solution (BSS Plus; Alcon or Opticosol solution; Reyon Pharm, Seoul, Korea). Throughout the surgery, ophthalmic viscoelastic material (15 mg/mL, Kukje Hyaluronate-I Inj; KUKJE, Geonggi, Korea) was utilized and subsequently removed through irrigation/aspiration (I/A) at the conclusion of the procedure. To ensure complete removal of viscoelastic substances, the eye was thoroughly irrigated via I/A for a duration of 30 s.

To ensure closure of the corneal incision, 8–0 polyglactin (Vicryl, Ethicon, Somerville, NJ) or 8–0 nylon (Usiol, Lexington, KY) sutures were used in a simple interrupted pattern with a spatula needle. At the conclusion of the surgery, dexamethasone (2 mg/eye; JEIL, Seoul, Korea) and cefazolin (50 mg/eye; Chongkundang) were subconjunctivally administered. In order to monitor postoperative IOP, measurements were taken immediately after the surgery and at 4-h intervals for a duration of 48 h.

As part of the postoperative regimen, topical steroids were prescribed, including prednisolone acetate 1% (applied every 6 h for 2 weeks, followed by a gradual reduction over 2 to 4 weeks) or neomycin-polymyxin B sulfate-dexamethasone (applied every 6 h for 2 weeks, followed by a gradual reduction over 4 weeks). Additionally, topical antibiotics such as moxifloxacin or ofloxacin were used (applied every 6 h for 2 weeks, and then every 8–12 h for 2 to 3 weeks). For systemic administration, antibiotics in the form of amoxicillin-clavulanate were given orally every 12 h for a duration of 2 to 3 weeks. Furthermore, systemic steroids in the form of prednisolone were administered orally at a dosage of 0.5 mg/kg every 12 h for 1 to 2 weeks, gradually tapering over 2 to 4 weeks.

### 2.4 Statistical analysis

The statistical analyses were conducted using SPSS software (version 17.0; SPSS Inc., Chicago, IL, United States). To account for co-variates such as breed and sex, we utilized the Chi-square (χ^2^) test to prove that there were no significant differences between groups for these categories. For age and body weight, a t-test was employed to demonstrate no significant group differences, ensuring a robust analysis by factoring in these important variables. ANOVA was then employed to compare the normal eye, cataract, and post-operative groups, with post-hoc analyses conducted using Fisher’s exact test for specific comparisons. The NSM and STC groups were compared using a t-test. A single linear regression analysis was performed for all test eyes to investigate the relationship between intraocular pressure (IOP), measured concurrently with the ophthalmic examination and UBM testing, and each parameter.

This meticulous approach in controlling for potential confounding factors such as breed, sex, age, and body weight enhances the integrity of our findings. The level of statistical significance was set at *p* < 0.05, *p* < 0.01, *p* < 0.001, and *p* < 0.0001, denoted by *, **, ***, and ****, respectively. For convenience in the figures, asterisks are used, while the actual *p*-values are reported in the text.

## 3 Results

### 3.1 Canine characteristics

#### 3.1.1 Normal eye group

In the normal eye group, a total of 13 eyes from 7 dogs were included in the study. Among these, 6 dogs were purebred and 1 dog was a mixed breed. The most common breeds were the Poodle, Shih Tzu and Maltese. The group consisted of 4 male and 3 female dogs. The average age of the dogs in this group was 8.50 ± 4.87 years (ranging from 1.5 to 15 years), with an average weight of 5.46 ± 2.46 kg (ranging from 2.85 to 9.80 kg).

#### 3.1.2 Cataract group

The cataract group consisted of 32 eyes from 20 dogs. Among these eyes, 14 were classified as incipient, 12 as immature, and 6 as mature cataracts. The majority of dogs in this group were purebred, with the most common breeds being Poodle, Bichon Frise, and Maltese. There were 8 male and 12 female dogs included in the study. The mean age of the dogs in this group was 8.25 ± 3.00 years (ranging from 2.2 to 13.0 years), and the average weight was 5.01 ± 2.40 kg (ranging from 1.80 to 9.80 kg).

#### 3.1.3 Post-phaco group

The post-phaco group included 12 eyes from 10 dogs, excluding patients with glaucomatous eyes. Before the surgery, the cataract stages of the included eyes were categorized as five immature and seven mature. Among the dogs in this group, 10 were purebred, with Maltese, Bichon Frise, and Poodle being the most common breeds. The group consisted of 3 male dogs and 7 female dogs. The mean age of the dogs in this group was 9.59 ± 2.71 years (ranging from 5.28 to 12.4 years), and their average weight was 4.50 ± 1.94 kg (ranging from 2.4 to 8.1 kg). There were no significant differences in age, weight, or breed distribution between the groups.

### 3.2 Ciliary muscle parameter changes according to cataract maturation

**Figure 2:**
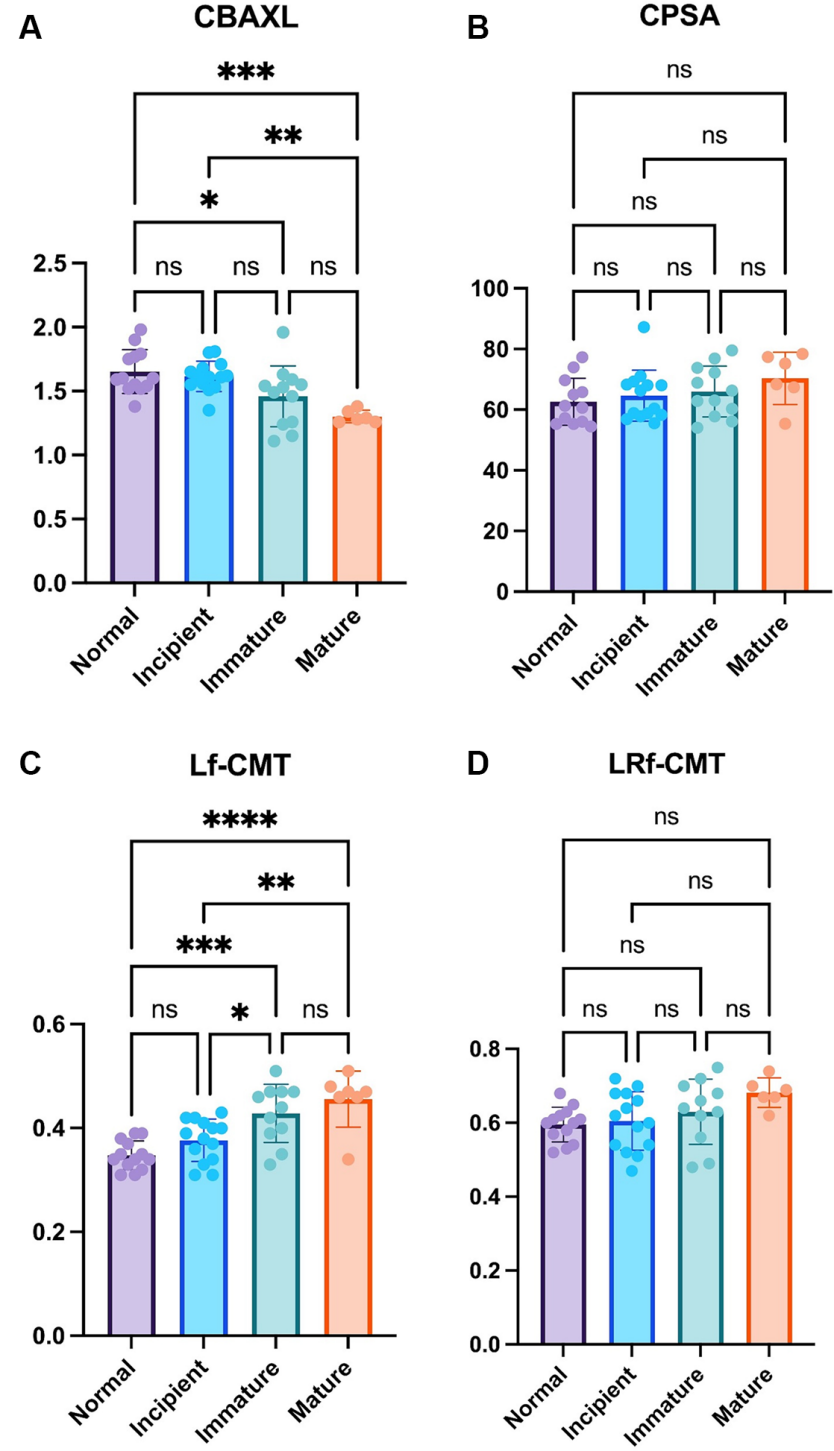
Comparison of the normal eye, incipient, immature and mature cataract **(A)** Axial length of the ciliary body (CBAXL), **(B)** ciliary process-sclera angle (CPSA), **(C)** longitudinal fiber of ciliary muscle thickness (Lf-CMT), and **(D)** longitudinal and radial fiber of ciliary muscle-choroid thickness (LRf-CMT) in the normal eye (*n* = 13), incipient cataract (*n* = 14), immature cataract (*n* = 12), and mature cataract (*n* = 6). The results showed that CBAXL tended to decrease as cataract progressed, with significant differences observed between the normal eye and immature eye, normal eye and mature eye, and incipient eye and mature eye groups. CPSA exhibited an overall increase, but no significant differences were found among the cataract stages. Lf-CMT showed a progressive increase with cataract advancement, with significant differences observed between the normal eye and immature eye, normal eye and mature eye, incipient eye and immature eye, and incipient eye and mature eye groups. LRf-CMT also demonstrated a tendency to increase, but no significant differences were observed among the cataract stages. The level of statistical significance was set at *p* < 0.05, *p* < 0.01, *p* < 0.001, and *p* < 0.0001, denoted by *, **, ***, and ****.

The results of the study showed that as the stage of cataract progressed, CBAXL tended to decrease overall. Statistically significant differences were observed between the normal eye and immature eye, normal eye and mature eye, and incipient eye and mature eye groups (1.65 ± 0.17 mm vs. 1.46 ± 0.24 mm, 1.65 ± 0.17 mm vs. 1.30 ± 0.05 mm, and 1.62 ± 0.12 mm vs. 1.46 ± 0.24 mm, respectively; *p* < 0.05, *p* < 0.001 and *p* < 0.01, respectively; Figure 2A). This indicates that as the cataract progresses, the shape of the ciliary body tends to change toward relaxation. In terms of CPSA, there was an overall tendency to increase as the stage of cataract progressed (Figure 2B). However, no significant difference was observed among the different stages of cataract. This suggests that the changes in CPSA were not significantly associated with the progression of cataract.

Regarding Lf-CMT, it showed an overall tendency to increase as the stage of cataract progressed. Statistically significant differences were found between the normal eye and immature eye, normal eye and mature eye, incipient eye and immature eye, and incipient eye and mature eye groups (0.35 ± 0.03 mm vs. 0.43 ± 0.06 mm, 0.35 ± 0.03 mm vs. 0.46 ± 0.05 mm, 0.38 ± 0.04 mm vs. 0.43 ± 0.06 mm and 0.38 ± 0.04 mm vs. 0.46 ± 0.05 mm, respectively; *p* < 0.001, *p* < 0.0001, *p* < 0.05 and *p* < 0.01, respectively; Figure 2C). These findings indicate that the longitudinal fibers of the ciliary muscle thickened as the cataract advanced. LRf-CMT, there was an overall tendency to increase with the progression of cataract (Figure 2D). However, no significant difference was observed among the different stages of cataract, suggesting that the changes in LRf-CMT were not significantly related to the stage of cataract.

Overall, the study revealed that the ciliary body undergoes changes in terms of axial length and ciliary muscle thickness as cataract progresses. These findings contribute to understanding the structural alterations that occur in the ciliary body during the development and progression of cataract.

### 3.3 Ciliary muscle changes in phacoemulsification STC and NSM groups

**Figure 3:**
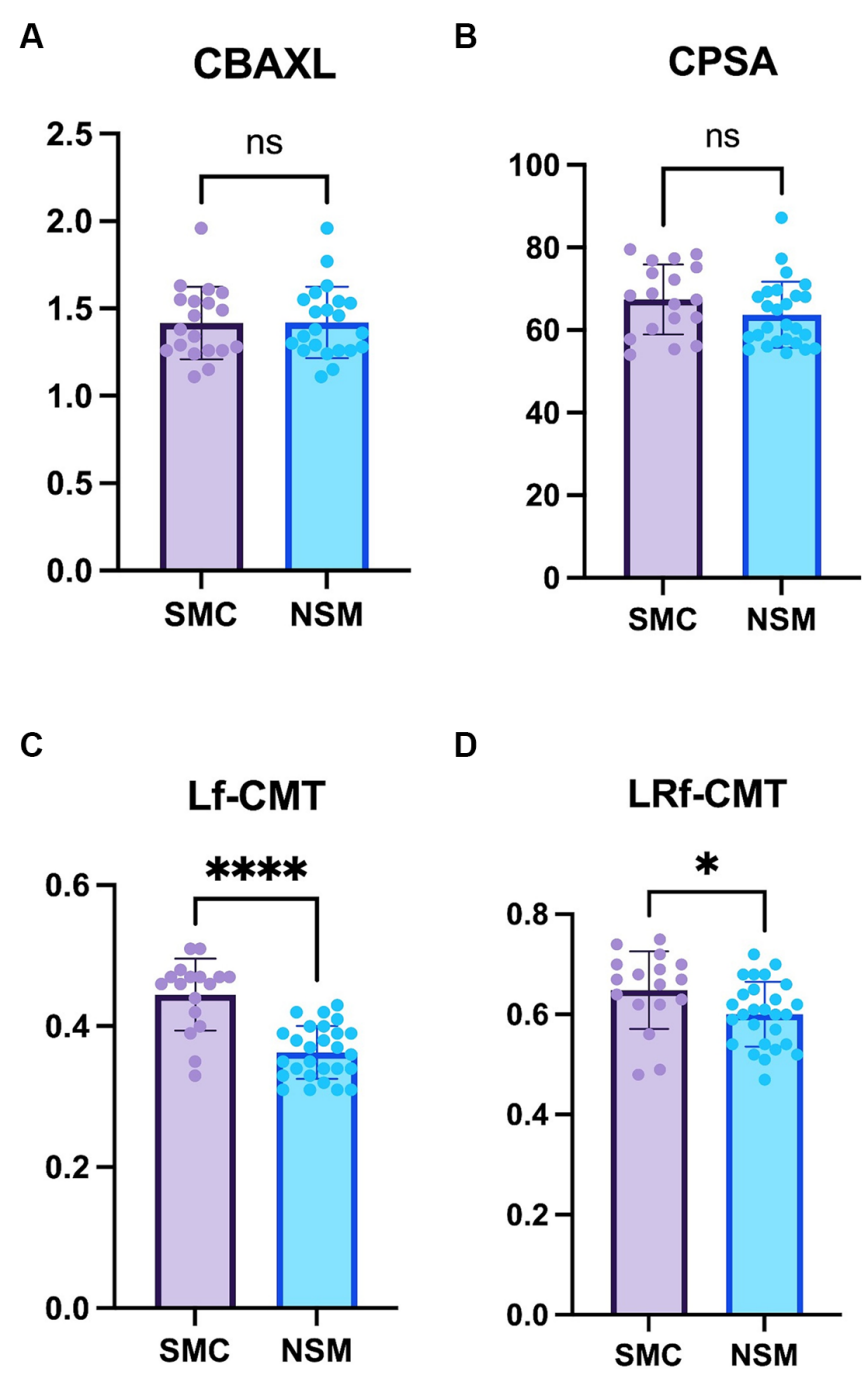
Comparison of the Non-surgical management and Surgical treatment candidate groups **(A)** Axial length of the ciliary body (CBAXL), **(B)** ciliary process-sclera angle (CPSA), **(C)** longitudinal fiber of ciliary muscle thickness (Lf-CMT), and **(D)** longitudinal and radial fiber of ciliary muscle-choroid thickness (LRf-CMT) in the Non-surgical management (NSM) group (*n* = 27) and Surgical treatment candidate (STC) groups (*n* = 18) undergoing phacoemulsification. The study revealed no significant differences between the two groups in CBAXL and CPSA. Regarding Lf-CMT, the STC group exhibited higher values compared to the NSM group. Also, significant difference was observed in LRf-CMT between the two groups. The level of statistical significance was set at *p* < 0.05, *p* < 0.01, *p* < 0.001, and *p* < 0.0001, denoted by *, **, ***, and ****.

The study revealed no significant differences in both CBAXL and CPSA between the groups with STC and those with NSM (Figures 3A,B). These findings suggest that the overall shape of the ciliary body remains consistent between the two groups.

Regarding the Lf-CMT, the study revealed that this value was significantly greater in the STC group compared to the NSM group (0.44 ± 0.05 mm vs. 0.36 ± 0.04 mm, respectively; *p* < 0.0001; Figure 3C). This suggests that the thickness of the longitudinal fibers experiences more notable changes in the STC group, likely as a result of cataract progression. Similarly, for the LRf-CMT, the value was found to be significantly higher in the STC group compared to the NSM group (0.65 ± 0.08 mm vs. 0.60 ± 0.06 mm; *p* < 0.05; Figure 3D).

Overall, the findings indicate significant differences in the thickness of the ciliary body between the STC and NSM groups. These observations contribute to our understanding of the structural changes that occur in the ciliary body in relation to cataract progression.

### 3.4 Ciliary muscle changes in normal eye, cataract, and post-phaco group

**Figure 4:**
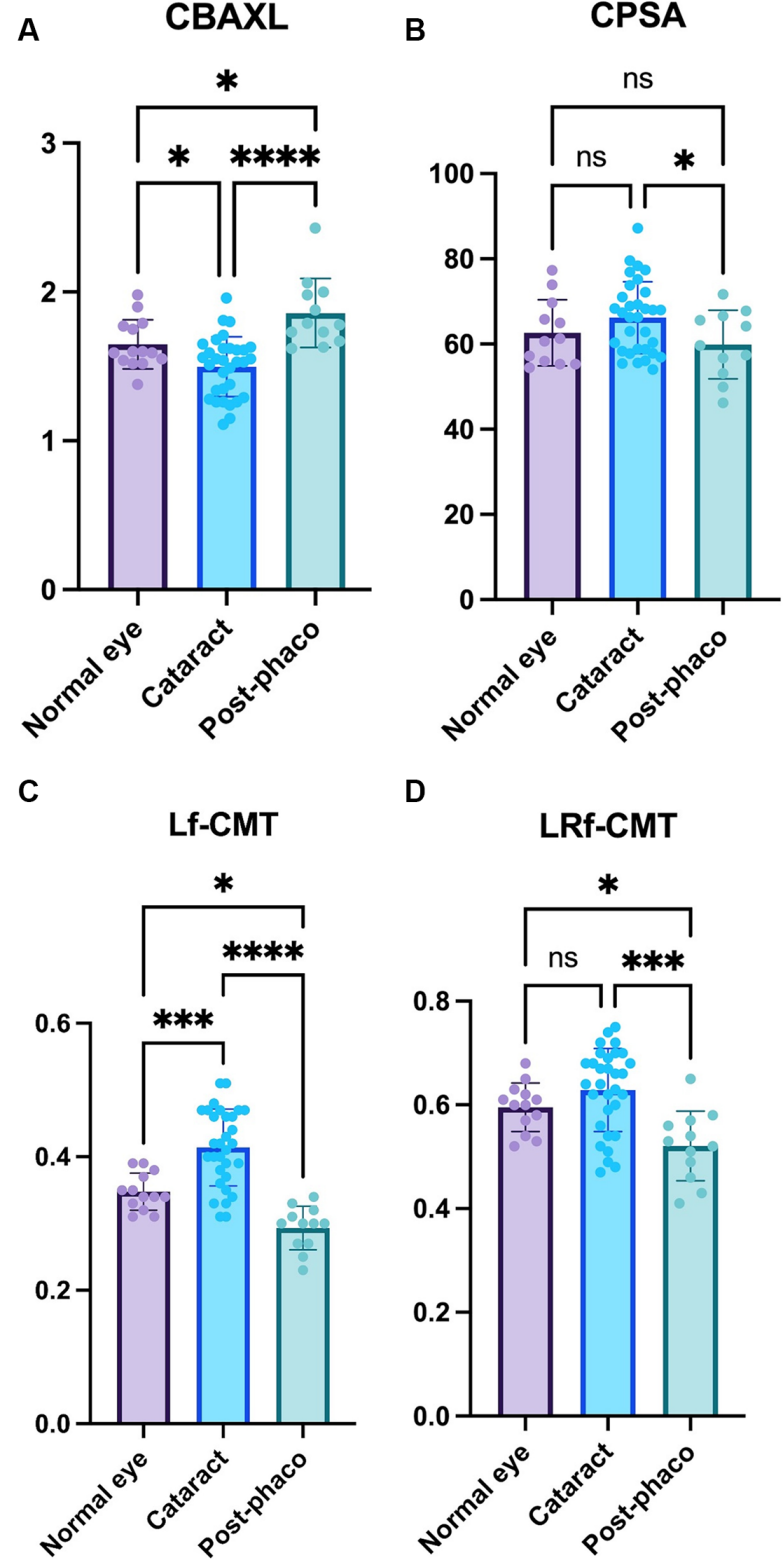
Comparison of the normal, cataract, and post-phaco groups **(A)** Axial length of the ciliary body (CBAXL), **(B)** ciliary process-sclera angle (CPSA), **(C)** longitudinal fiber of ciliary muscle thickness (Lf-CMT), and **(D)** longitudinal and radial fiber of ciliary muscle-choroid thickness (LRf-CMT) in the normal eye (*n* = 13), cataract (*n* = 32), and post-phaco groups (*n* = 12). The study revealed significant differences among the groups in CBAXL, CPSA, Lf-CMT, and LRf-CMT. The cataract group exhibited a smaller CBAXL value compared to the normal eye group, while the post-phaco group showed a significant increase in CBAXL compared to the cataract group. Additionally, the CBAXL value was higher in the post-phaco group than in the normal eye group. In terms of CPSA, it was found to be significantly smaller in the post-phaco group compared to the cataract group. Regarding Lf-CMT, the cataract group demonstrated an increase compared to the normal eye group, while the post-phaco group exhibited a significant decrease compared to the cataract group. Moreover, the post-phaco group showed a significant decrease in Lf-CMT compared to the normal eye group. For LRf-CMT, it was observed to be significantly smaller in the post-phaco group compared to both the cataract and normal eye groups. The level of statistical significance was set at *p* < 0.05, *p* < 0.01, *p* < 0.001, and *p* < 0.0001, denoted by *, **, ***, and ****.

Regarding CBAXL, the cataract group exhibited a significantly smaller value compared to the normal eye group (1.50 ± 0.20 mm vs. 1.65 ± 0.17 mm; *p* < 0.05). Furthermore, the post-phaco group showed a significant increase in CBAXL compared to the cataract group (1.86 ± 0.23 mm vs. 1.50 ± 0.20 mm; *p* < 0.0001). Additionally, the CBAXL value was significantly higher in the post-phaco group than in the normal eye group (1.86 ± 0.23 mm vs. 1.65 ± 0.17 mm; *p* < 0.05; Figure 4A). In terms of CPSA, it was found to be significantly smaller in the post-phaco group compared to the cataract group (59.89 ± 8.04 vs. 66.20 ± 8.44; *p* < 0.05; Figure 4B).

Regarding Lf-CMT, the cataract group demonstrated a significant increase compared to the normal eye group (0.41 ± 0.06 mm vs. 0.35 ± 0.03 mm; *p* < 0.001), while the post-phaco group exhibited a significant decrease compared to the cataract group (0.29 ± 0.03 mm vs. 0.41 ± 0.06 mm; *p* < 0.0001). Moreover, the post-phaco group showed a significant decrease in Lf-CMT compared to the normal eye group (0.29 ± 0.03 mm vs. 0.35 ± 0.03 mm; *p* < 0.05; Figure 4C). For LRf-CMT, it was observed to be significantly smaller in the post-phaco group compared to the cataract group (0.52 ± 0.07 mm vs. 0.63 ± 0.08 mm; *p* < 0.001) and significantly smaller in the post-phaco group compared to the normal eye group (0.52 ± 0.07 mm vs. 0.60 ± 0.05 mm; *p* < 0.05; Figure 4D).

Overall, the results indicate that the CBAXL and CPSA values differ significantly between the cataract and post-phaco groups. Furthermore, Lf-CMT and LRf-CMT show significant changes between the normal eye, cataract, and post-phaco groups. These findings suggest that the alterations in parameter values observed during the transition from the normal eye to cataract formation are reversed following phacoemulsification.

### 3.5 Associations between IOP and measured parameters

**Figure 5:**
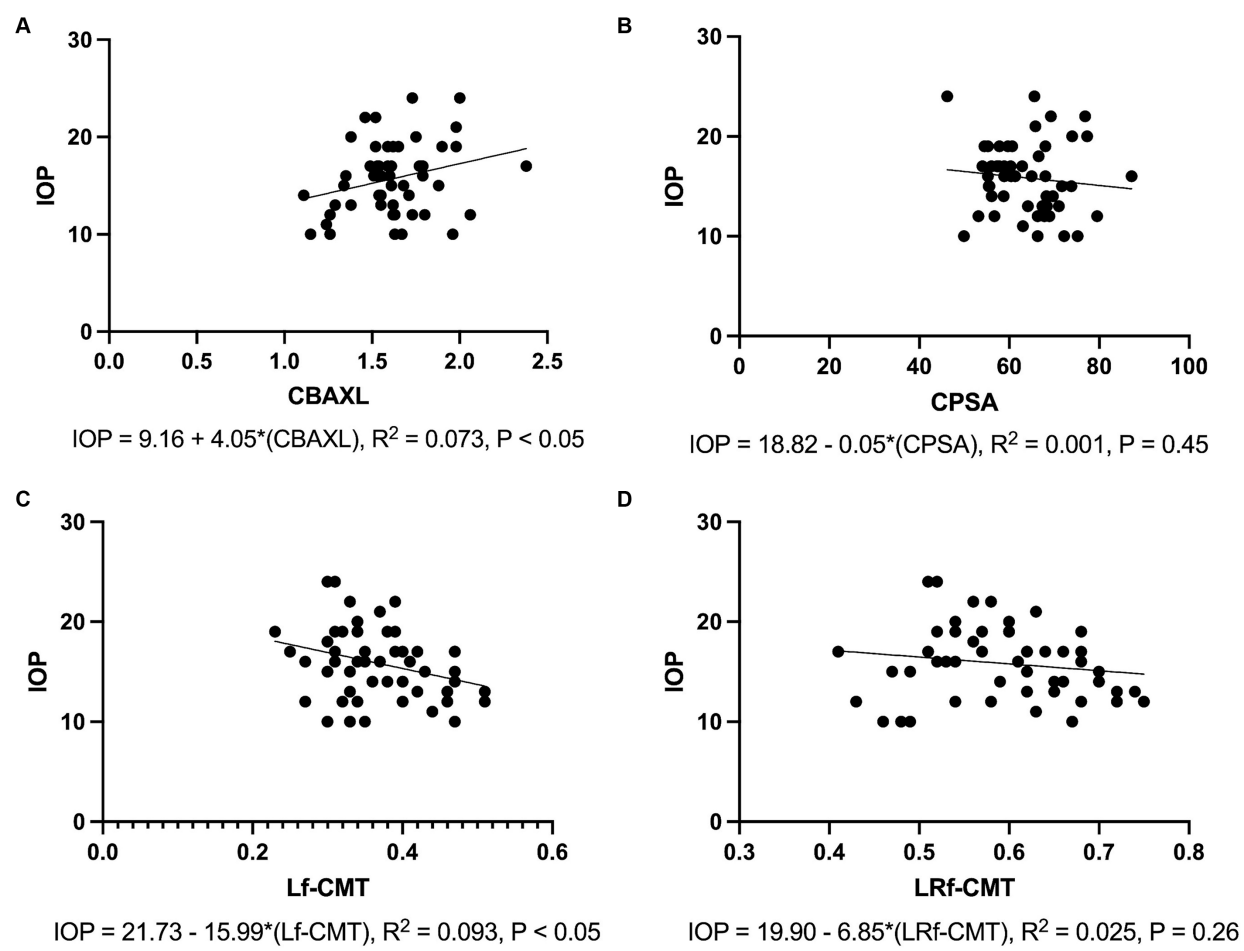
Associations between Intraocular Pressure and Measured Parameters **(A)** Positive association is observed between IOP and the axial length of the ciliary body (CBAXL), with the regression model accounting for 7.3% of the variance in IOP (*p* < 0.05). **(B)** No significant association is found between IOP and ciliary process-sclera angle (CPSA), with the regression model explaining only 0.1% of the variance in IOP (*p* = 0.45). **(C)** A negative association is noted between IOP and the longitudinal fiber of ciliary muscle thickness (Lf-CMT), with the regression model accounting for 9.3% of the variance in IOP (*p* < 0.05). **(D)** No significant association is detected between IOP and the longitudinal and radial fiber of ciliary muscle-choroid thickness (LRf-CMT), with the regression model explaining only 2.5% of the variance in IOP (*p* = 0.26).

The results of the regression analysis revealed significant associations between IOP and the measured parameters. Specifically, there was a positive association between IOP and CBAXL (Figure 5A). The regression model accounted for 7.3% of the variance in IOP, and the association was statistically significant (*p* < 0.05). In contrast, a negative association was observed between IOP and CPSA (Figure 5B). However, the regression model explained only 0.1% of the variance in IOP, and the association was not statistically significant (*p* = 0.45).

Regarding the difference between Lf-CMT, a negative association was found with IOP. The regression model accounted for 9.2% of the variance in IOP, and the association was statistically significant (*p* < 0.05; Figure 5C). On the other hand, the difference between LRf-CMT showed a negative association with IOP. However, the regression model explained only 2.5% of the variance in IOP, and the association was not statistically significant (*p* = 0.26; Figure 5D).

Overall, these results indicate that CBAXL and Lf-CMT have significant associations with IOP, suggesting their potential relevance in understanding IOP changes.

## 4 Discussion

The purpose of this study was to investigate the morphological changes of the ciliary muscle before and after phacoemulsification, aiming to better understand the underlying causes of post-operative glaucoma. The ciliary muscle consists of three layers: longitudinal fibers, radial fibers, and circular fibers (26). To examine these morphological changes from different perspectives, we categorized the parameters into two distinct groups. The first group included CBAXL and CPSA, which indicate the function of the circular fibers and provide insights into the overall motion of the ciliary body (11, 14). The second group comprised Lf-CMT and LRf-CMT, representing the function of the longitudinal fibers and their association with unconventional outflow facility (3, 14). By analyzing these two parameter groups in dogs undergoing cataract surgery, specifically after phacoemulsification, we gained a comprehensive understanding of the morphological changes in the ciliary muscle and their potential contributions to the development of glaucoma.

This study serves as a continuation of our previous research, incorporating some of the same patients. Our goal in this investigation was to conduct a more detailed analysis of the ciliary body, with a particular focus on the ciliary muscle. Through this approach, our research has, for the first time, analyzed the effects of the ciliary body on the expansion and contraction of the ciliary cleft. To achieve this, we carefully selected images from the earlier dataset that clearly depicted the dome shape of the ciliary body. Although this study includes some of the same patients from our previous research, it is important to note that we used different photographs this time. Unlike the previous study, which primarily used images focusing on the ciliary cleft, the current investigation selected photographs emphasizing the ciliary body, even when involving the same patients. This deliberate selection aimed to ensure a thorough examination of the specific anatomical features of interest. By utilizing images with clear representations of these structures, we sought to enhance our understanding of the complexities of the ciliary body.

In this study, we aimed to diversify our group categorizations to enhance the multifaceted analysis of our data. Initially, we analyzed each parameter according to the maturity of the cataract to determine if there were any significant differences in the relaxation of the ciliary body across various stages. To overcome the limitations of comparisons between these finely divided first groups, we categorized the normal and incipient groups as NSM groups and the immature and mature groups as STC groups for cataract surgery. This allowed us to investigate whether there were significant changes in the ciliary body among the groups eligible for cataract surgery. Finally, by dividing the subjects into normal, cataract, and post-phaco groups, we aimed to ascertain the degree of difference in the ciliary body post-surgery compared to that in cataract-affected eyes, and how these compare to the normal group. This layered approach to grouping was designed to meticulously assess the impact of cataract maturity and surgical intervention on the ciliary body’s behavior.

Previous studies in humans have extensively investigated changes in the ciliary muscle in relation to accommodation. Findings from a study conducted on rhesus monkeys have demonstrated that the ciliary muscle undergoes anterointernal movement upon contraction, primarily mediated by the action of circular fibers. Additionally, compaction occurs in the longitudinal fibers, resulting in an overall reduction in thickness (13, 24, 27). These morphological changes in the ciliary muscle can be observed as an increase in CBAXL, indicating inward movement of the ciliary muscles, and a decrease in CPSA, reflecting anterior movement of the ciliary muscles (10, 11). Similarly, the contraction of the ciliary muscle is associated with a decrease in the value of Lf-CMT and LRf-CMT.

In our study, several key findings related to the motion of the ciliary body were observed. Firstly, there was a consistent pattern of diminishing CBAXL as the stage of cataract in dogs advanced. The cataract group showed significantly lower CBAXL values compared to those in the normal eye group. While the difference in CPSA among the cataract stage was not statistically significant, a trend was noted for CPSA values to increase with the progression of cataracts. These observations imply that as cataracts develop, the ciliary muscle tends to relax, leading to the outward movement of the ciliary body. Such findings align with prior research conducted in humans and support the hypotheses posited in our study (10, 28). As cataracts progress, an increase in lens thickness and a corresponding rise in zonular fiber tension occur, resulting in a biologically relaxed state of the ciliary body (10, 11, 29, 30).

The results following phacoemulsification revealed contrasting patterns of ciliary body motion compared to those observed in the presence of cataract. In the post-phaco group, the CBAXL value was higher than that in the cataract group, which, in turn, was higher than that in the normal group. Similarly, for CPSA, the post-phaco group exhibited smaller values compared to the cataract group. These findings indicate that the ciliary muscle undergoes contraction following phacoemulsification, resulting in inward and anterior movements of the ciliary body. These observations align with previous studies conducted in humans, where cataract extraction led to a decrease in lens thickness, potentially reducing zonular tension and facilitating improved ciliary body motion (10). Furthermore, it is noteworthy that the degree of ciliary muscle contraction in the post-phaco group was greater than that in the normal eye, likely due to the complete removal of the lens material. In the case of CPSA, the magnitude of change was comparatively smaller than that of CBAXL. This may be attributed to the posterior movement of the ciliary process caused by the posterior dropping of the lens capsule into the potential space created by lens extraction (31).

Based on our previous research findings, it is apparent that the movement of the ciliary body exerts a significant influence on the CC. The CC, which assumes a triangular shape, exists between the two distinct “leaves” of the ciliary muscle (8, 32–34). This structure, residing within the ciliary muscle, undergoes alterations in response to the motion of the ciliary body. Specifically, in the case of cataract, the CC expands when the ciliary muscle relaxes and the ciliary body moves outward and posteriorly. Conversely, during phacoemulsification, the contraction of the ciliary muscle causes the ciliary body to shift inward and anteriorly, resulting in the narrowing of the CC.

These findings present a contradiction to human studies, which demonstrate that ciliary muscle contraction leads to the widening of the trabecular meshwork (14). It is postulated that these discrepancies arise due to variations in the positioning of the trabecular meshwork between humans and dogs. In humans, the trabecular meshwork is situated within the scleral sulcus, with the anterior tendons of the ciliary muscle inserting into the outer portion of the corneoscleral meshwork and the juxtacanalicular meshwork (5, 35). Conversely, in dogs, the ciliary muscle directly influences the movement of the ciliary body, resulting in the presence of a CC between the “leaves” of the ciliary muscle (33, 34). Consequently, these anatomical distinctions contribute to the conflicting outcomes observed in humans and dogs.

The CC plays a pivotal role in the outflow of AH and has a significant impact on the regulation of IOP (36). The expansion and relaxation of the CC are influenced by the ciliary muscle. In this study, a regression analysis was conducted to explore the relationship between IOP and CBAXL. The results revealed a positive correlation between the two variables, suggesting that the movement of the ciliary muscle has some influence on IOP. However, it is important to note that this correlation, while statistically significant, only accounted for 7.3% of the variance in IOP. This implies that the control of IOP is not solely achieved through the manipulation of the CC via the contraction and relaxation of the ciliary muscle. Contraction of the ciliary muscle is known to facilitate the opening of radial collector channels and increase the conventional outflow pathway in human studies. (8, 37). Besides, it is crucial to acknowledge that various factors, such as AH production, arterial pressure, and age, can also impact IOP (38). Despite these complexities, the movement of the ciliary muscle is still considered to have a significant effect on IOP, highlighting its relevance in the overall regulation of ocular dynamics.

Secondly, we examined the ciliary muscle thickness. As the cataract stage advanced, there was a noticeable increase in the Lf-CMT values. In fact, most stages demonstrated statistically significant differences, with only a few exceptions. When comparing the STC group with the NSM group, the STC group exhibited significantly higher Lf-CMT values. Similar significant differences were also observed between the normal eye group and the cataract group. However, no significant difference was found in LRf-CMT when comparing groups based on cataract progression. These findings support the notion that the ciliary muscle tends to relax as the cataract progresses, which is consistent with the results obtained for CBAXL and CPSA. Specifically, the remarkable increase in thickness observed in the longitudinal fibers during relaxation is noteworthy when compared to the radial fibers.

After phacoemulsification, changes in ciliary muscle thickness exhibited a contrasting pattern compared to cataract disease. In the post-phaco group, both Lf-CMT and LRf-CMT values were smaller compared to the cataract group, which, in turn, were smaller than those of the normal group. As mentioned earlier, this can be attributed to the contraction of the ciliary muscle following the removal of lens material through phacoemulsification, leading to decreased tension in the zonules. Unlike the comparison with cataract patients, the difference in LRf-CMT values was statistically significant. This suggests that the radial muscle exhibits minimal relaxation in the presence of cataract, while the contraction effect due to phacoemulsification is more pronounced.

The ciliary muscle serves as the primary site of resistance for unconventional outflow pathways (39–41). These pathways, including uveoscleral, uveovortex, and uveolymphatic routes, traverse the interstitial spaces of the ciliary muscle (2). Other routes such as the iridal and corneal routes are considered to be negligible in terms of unconventional outflow (42). Among these outflows, the path through the ciliary muscle is particularly crucial as it acts as a rate-limiting step (4). As AH passes through the interstitial spaces between ciliary muscles, the contraction and relaxation of the ciliary muscles, i.e., their tone, exert the greatest influence on AH flow (3). Contraction of the ciliary muscle causes individual muscle fibers to swell and compact, reducing the interstitial space and increasing AH resistance (41). For example, in the study by Bill et al. on human medicine, it was observed that in eyes which did not receive any medication for a period of 48 h, the uveoscleral flow constituted 4 and 14% of the total outflow. When atropine is administered, inducing relaxation of the ciliary muscle, uveoscleral flow accounts for approximately 4 to 27% of the total aqueous humor outflow. This is in stark contrast to the effects of pilocarpine administration, which causes ciliary muscle contraction and leads to a significantly reduced uveoscleral flow, ranging from 0 to 3%. This research underscores how changes in ciliary muscle tone can significantly alter uveoscleral outflow (43, 44).

Based on this perspective, in this study, the rapid decrease in Lf-CMT and LRf-CMT observed after phacoemulsification indicates a reduction in the interstitial space of the ciliary muscle, resulting in a significant inhibition of AH flow. Moreover, patients with cataract exhibited relatively larger Lf-CMT, indicating a wider interstitial space of the ciliary muscle and reduced resistance to AH flow. Regarding the difference in Lf-CMT, a negative association with IOP was observed. The regression model accounted for 9.3% of the variance in IOP, and the association was statistically significant. Considering that unconventional outflow contributes to approximately 15% of total outflow in dogs, the 9.3% contribution of Lf-CMT is considered to be substantial. In contrast, the regression analysis of LRf-CMT and IOP did not yield statistically significant results. This may be because the flow of AH predominantly occurs through the longitudinal fibers rather than the radial fibers (39). In previous studies utilizing conventional perfusion techniques, it has been demonstrated that the flow of AH from the ciliary muscle occurs through the interstitial spaces between the longitudinal ciliary muscle bundles and into the supraciliary and suprachoroidal spaces (45–47).

Simply measuring the thickness of the ciliary muscle does not provide a complete understanding of AH passage through the ciliary muscle. The connective tissue present within the interstitial space of the ciliary muscle is known to impede the flow of AH (8, 40, 48). Studies conducted in humans have reported an increase in the proportion of connective tissue in this area, from approximately 20% in individuals aged 30–40 to over 50% in individuals aged 60 and above (12, 49). This increase is likely to reduce the amount of AH passing through the uveoscleral route. Similar studies in veterinary medicine have revealed that beagles with advanced glaucoma tend to accumulate melanophores and develop an extracellular matrix abundant in elastic fibers within their ciliary body. These deposits may contribute to elevated unconventional outflow resistance and subsequently reduce unconventional outflow in these animals (50). However, since there was no statistically significant age difference between the groups in this study, it is assumed that the scale of connective tissue did not differ significantly. Therefore, the thickness of the ciliary muscle is considered an important factor influencing unconventional outflow.

In our previous paper, we proposed that prophylactic medications targeting AH production, such as beta-blockers and carbonic anhydrase inhibitors, may be helpful in reducing IOP elevation caused by CC narrowing after phacoemulsification (28). Furthermore, based on the findings of this study, it is estimated that drugs like phenylephrine, which relax the ciliary muscle by acting on the parasympathetic nervous system, could potentially be beneficial in this context (51). Further research related to this topic will be needed to investigate and validate the potential efficacy of these medications.

This study acknowledges certain limitations. The accurate distinction of individual ciliary muscle fibers using UBM is challenging (24). Therefore, the location of each fiber was estimated based on anatomical knowledge. The measurements were conducted assuming that longitudinal fibers are positioned just beneath the sclera and run parallel to the iris, radial fibers are situated between longitudinal and circular fibers, and circular fibers originate from the posterior iris (8, 26, 52). Although these parameters were obtained through estimation, this study is significant as the first veterinary research that anatomically classifies ciliary muscles and explains their individual roles. Furthermore, it holds great importance to establish correlations between these anatomical components and the flow of AH, as well as to identify potential causes of glaucoma following phacoemulsification.

Another limitation of this study is the exclusion of hypermature cataracts. In hypermature cataracts, the lens begins to shrink, and the lens capsule appears wrinkled. At this stage, lens-induced uveitis (LIU) often occurs (53). This research focused on the hardening and thickening of the lens, along with the resultant changes in zonular tension and the effects on the ciliary body. Therefore, it is hypothesized that in cases of hypermature cataracts, the level of zonular tension might be lower than in mature cataracts, and the contraction of the ciliary body might be more pronounced. Additionally, LIU could lead to relaxation of the ciliary muscle and a consequent decrease in IOP (54). To avoid confusion in the research findings due to these multiple interfering factors, hypermature cataracts were excluded from this study.

A further limitation of this research is the utilization of 0.5% tropicamide. Known for its potential to elevate IOP by inducing contraction of the ciliary cleft, tropicamide also serves as a cycloplegic agent, paralyzing the ciliary muscle (35, 55). Despite this, the consistent application of tropicamide across all participants in this study likely helped to minimize the impact of varying iris sphincter responses due to light levels, thus contributing to the uniformity of the UBM measurements. It is also noted that the 0.5% concentration of tropicamide used is not considered potent enough to achieve full cycloplegia (56).

**Figure 6:**
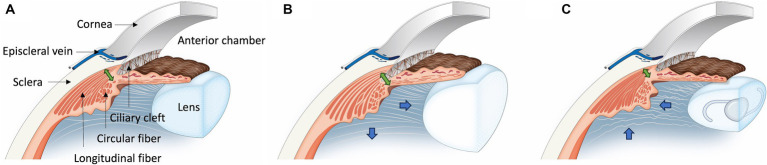
Illustration of ciliary muscle changes after phacoemulsification in cataract dogs. **(A)** In the Normal Eye Group, the ciliary muscle maintains its normal position. **(B)** For the Cataract Eye, there is an outward and posterior movement (Blue arrow) of the ciliary muscle compared to the normal eye, leading to an expansion of the ciliary cleft and an increase in the longitudinal fiber thickness (Green double arrow). Additionally, there is an increase in the interstitial space between the longitudinal ciliary muscle fibers. **(C)** In the Post-Phaco Eye group, the ciliary muscle shifts inward and anteriorly (Blue arrow), more pronounced than in the cataract eye, resulting in a narrowing of the ciliary cleft. Compared to both the cataract group and the normal eye, the longitudinal fiber thickness is thinner (Green double arrow). Furthermore, there is a decrease in the interstitial space between the longitudinal ciliary muscle fibers.

Based on the findings of this study, two key perspectives can be considered: ciliary muscle motion and ciliary muscle thickness (Figure 6). Firstly, in patients with cataract, the relaxation of the ciliary muscle leads to the expansion of the CC and an increase in ciliary muscle thickness. Conversely, after phacoemulsification, the ciliary muscle contracts, causing the CC to collapse and the ciliary muscle thickness to decrease. These changes suggest an increase in resistance to both conventional and unconventional outflow following phacoemulsification, which could potentially contribute to the development of glaucoma.

## Data availability statement

The original contributions presented in the study are included in the article, further inquiries can be directed to the corresponding author.

## Ethics statement

The animal studies were approved by The Institutional Animal Care and Use Committee Of Chungbuk National University. The studies were conducted in accordance with the local legislation and institutional requirements. Written informed consent was obtained from the owners for the participation of their animals in this study.

## Author contributions

DK: Conceptualization, Data curation, Formal analysis, Investigation, Methodology, Writing – original draft, Writing – review & editing. S-EP: Investigation, Resources, Writing – original draft. JH: Investigation, Resources, Writing – original draft. NK: Investigation, Resources, Writing – original draft. JJ: Investigation, Resources, Writing – original draft. K-MP: Supervision, Writing – review & editing.
